# Emergence of Respiratory *Streptococcus agalactiae* Isolates in Cystic Fibrosis Patients

**DOI:** 10.1371/journal.pone.0004650

**Published:** 2009-02-27

**Authors:** Vera Eickel, Barbara Kahl, Beatrice Reinisch, Angelika Dübbers, Peter Küster, Claudia Brandt, Barbara Spellerberg

**Affiliations:** 1 Institute of Med. Microbiology and Hygiene University of Ulm, Ulm, Germany; 2 Institute of Med. Microbiology, University Clinic Münster, Münster, Germany; 3 Department of Pediatrics, University Clinic Muenster, Muenster, Germany; 4 Department of Pediatrics, Clemenshospital Muenster, Muenster, Germany; 5 Institute of Medical Microbiology University Hospital Frankfurt, Frankfurt, Germany; Columbia University, United States of America

## Abstract

*Streptococcus agalactiae* is a well-known pathogen for neonates and immunocompromized adults. Beyond the neonatal period, *S. agalactiae* is rarely found in the respiratory tract. During 2002–2008 we noticed *S. agalactiae* in respiratory secretions of 30/185 (16%) of cystic fibrosis (CF) patients. The median age of these patients was 3–6 years older than the median age CF patients not harboring *S. agalactiae*. To analyze, if the *S. agalactiae* isolates from CF patients were clonal, further characterization of the strains was achieved by capsular serotyping, surface protein determination and multilocus sequence typing (MLST). We found a variety of sequence types (ST) among the isolates, which did not substantially differ from the MLST patterns of colonizing strains from Germany. However serotype III, which is often seen in colonizing strains and invasive infections was rare among CF patients. The emergence of *S. agalactiae* in the respiratory tract of CF patients may represent the adaptation to a novel host environment, supported by the altered surfactant composition in older CF patients.

## Introduction

*Streptococcus agalactiae* (group B streptococci, GBS) is an important human pathogen causing invasive disease in neonates and immunocompromized adult patients. While the microorganism is still the major cause of invasive bacterial infections in newborns, cases among newborns declined during the last years due to effective peripartal antibiotic prophylaxis. Nowadays over 80% of invasive infections are observed in patients older than 18 years and invasive disease in older patients appears to become more prevalent. Interestingly in the years 2003, 2004 and 2005 more adult patients died following invasive *S. agalactiae* infections than invasive *S. pyogenes* disease (http://www.cdc.gov/ncidod/dbmd/abcs/survreports/gbs05.pdf). These data suggest that the epidemiology of *S. agalactiae* is changing and that the bacteria are adapting to novel environments within the human host.

In contrast to closely related bacterial species like *Streptococcus pyogenes*, *S. agalactiae* is only rarely seen as a colonizer in respiratory secretions from pediatric patients beyond the neonatal period [1], [2]. Investigations concerning the presence of *S. agalactiae* in the respiratory tract of cystic fibrosis patients have to our knowledge not been published. Microorganisms most frequently seen in the respiratory secretions of patients suffering from cystic fibrosis (CF) are *Pseudomonas aeruginosa*, *Staphylococcus aureus* and *Haemophilus influenzae* [3], [4]. The occurrence of beta hemolytic streptococci is rarely reported in CF patients. One study recovered 6 isolates from the analysis of 258 CF respiratory tract specimens [5]. A more recent comprehensive investigation of 465 CF patients for colonizing gram positive microorganisms isolated *S. pyogenes* in 4 samples but did not find a single patient colonized by *S. agalactiae* [4].

Traditional subtyping methods for *S. agalactiae* rely on antibody determination of the capsular serotypes and surface protein structures. During the last years the repertoire of subtyping methods for *S. agalactiae* have been expanded by DNA sequence based methods like multi locus sequence typing (MLST) [6]. The technique has become one of the most powerful tools to characterize bacterial populations. It offers major advantages in comparison to older methods. The results are unambiguous, stable and the profiles of strains can be easily compared between different laboratories without the need to analyze strains of interest in one experiment. Moreover the data are comparable via the internet between different laboratories, which facilitates population analyzes worldwide. During the last years hundreds of invasive and colonizing *S. agalactiae* strains have been characterized with this method und today more than 380 MLST sequence types for *S. agalactiae* are recognized. Evolutionary relationships in the population structure of *S. agalactiae* can be analyzed with the eBURSTV3 program which identifies clonal complexes based on variations in the allelic MLST profiles of analyzed strains and allows a graphic representation of genetic relatedness [7].

The detection of *S. agalactiae* isolates in 16% of the patients from a population of 185 CF patients during 2002–2008 was surprising and to rule out a local outbreak, the molecular epidemiology of the strains was investigated. Furthermore the CF strains were compared to the general population structure of German *S. agalactiae* strains. For this purpose, 72 colonizing strains that were collected during a recent German colonization study of healthy adults [8] were characterized by MLST.

## Materials and Methods

### Processing of CF respiratory tract specimens

Respiratory specimens from CF patients were collected during clinical visits between 2002 and 2008. Samples were sent in from two different clinics spezialized on the treatment of CF patients in the region of Münster, Germany. Sputa were processed as follows: 500 µl sputum was mixed with 500 µl 0.5% acetylcystein (Merck), vortexed, incubated for 30 min at 35°C and homogenized by vortexing. 100 µl of the liquidified sputum was plated on Columbia blood and Endo agar for semiquantitative analysis. Primary cultures were performed on Columbia (Becton Dickinson, Heidelberg, Germany) sheep blood (Oxoid, Wesel, Germany) agar for Gram-positive cocci, on Endo agar (Merck, Darmstadt, Germany) for Gram-negative rods for 48 h at 35°C and on chocolate agar (Mast, Reinfeld, Germany) for *Haemophilus influenzae* for 24–48 h at 35°C under 5% CO_2_. Additionally, specimens were cultured in dextrose broth to enrich bacterial growth and streaked on blood and Endo agar after 48 h. Species identification was performed by respective standard procedures and the VITEK2 (bioMerieux, Marcy l'Etoile, France).

### Molecular typing of capsular antigens and surface proteins

Capsular serotypes of analyzed strains were determined by PCR of *cps* genes for serotypes Ia, Ib, III, IV, V and VI and DNA sequencing for serotype II strains was carried out as described by Kong F, et al. [9]. To determine the surface proteins of the strains a multiplex PCR reaction was carried out as described in [10]. For strains that did not yield any results in the multiplex PCR reaction, the PCR was repeated using the primer pairs specific for one surface protein in a single reaction mixture.

### Multi locus sequence typing

Multi locus sequence typing (MLST) of the *S. agalactiae* strains from CF patients and the 72 colonizing isolates was carried out as described by [6]. The colonizing strains were collected in a previous study [8], they originated from either vaginal or rectal specimens. Primers as listed in [6] were obtained from a commercial supplier (Thermo Hybaid, Ulm, Germany). Genomic bacterial DNA of *S. agalactiae* was isolated by using the QiaAmp DNA kit (Qiagen, Hilden, Germany) as described by the manufacturer. PCR conditions to amplify the *adhP*, *pheS*, *atr*, *glnA*, *sdhA*, *glcK* and *tkt* genes were set as described previously [6]. Sequencing of the generated PCR products was performed on an ABI Prism 310 Genetic Analyser (ABI Prism Biosystems, Warrington, UK) according to the instructions of the manufacturer. For analysis of the obtained nucleotide sequences and assignment of MLST profiles the following website: http://pubmlst.org/sagalactiae/ was used. Burst analysis to reveal the relationship of MLST sequence types and to analyze clonal complexes was carried out with the eBURSTV3 version that is accessible under: http://eburst.mlst.net.

### Antimicrobial susceptibility determination

For all CF *S. agalactiae* isolates, minimal inhibitory concentrations (MIC) of antibiotics were determined. The Merlin MICRONAUT system was used to determine MIC values for penicillin, ampicillin, cefuroxime, ceftriaxone, erythromycin, clarithromycin, clindamycin, doxycycline and moxifloxacin. Standard susceptibility testing was performed after overnight culture on blood agar plates as detailed in [11]. MIC values for gentamicin, vancomycin, rifampicin and levofloxacin were determined by E-test in accordance with the manufacturer's instructions (AB-Biodisk, Solna Sweden).

## Results

### Clinical characteristics of *S. agalactiae* positive CF patients

From 16 of the 30 *S. agalactiae* positive CF patients bacterial isolates for analysis by MLST were available. All of the strains displayed a typical *S. agalactiae* phenotype. From the 16 patients a total of 29 *S. agalactiae* strains were recovered and analyzed. Among these strains there were 19 unique *S. agalactiae* strains and 10 strains that turned out to be duplicates of previous isolates in patients that were colonized for a longer period of time. Clinical characteristics of these patients are shown in Table 1. The female to male ration among the patients was 50%. Only 1 patient of the 16 was younger than 10 years at the time *S. agalactiae* was first detected. While the median age of the *S. agalactiae* positive CF patients was 16,5 (range 10–22) and 22,5 (range 19–44) respectively at the two different clincis at the end of 2008, the median age of the general CF population not harboring *S. agalactiae*, at this clinics was 13,6 (range 1,3–35,8) and 16,22 (range 0,7–50). To assess the potential clinical implications of *S. agalactiae* in the respiratory tract of CF patients, we obtained the information, if *S. agalactiae* isolation occurred during a routine visit or during a visit due to an exacerbation of clinical symptoms. For two of the patients it was not possible to gather this information retrospectively. For the other patients, 24 clinical visits were recorded in connection with positive *S. agalactiae* isolation, 12 of these visits occurred due to an exacerbation of symptoms, while the others were scheduled routine visits. In most cases *S. agalactiae* was not the sole bacterial isolate from the sputum samples. The concomitant pathogenic bacteria that were isolated are listed in Table 1. Interestingly in 10 of 16 patients *Staphylococcus aureus* was found, while only 4 patients harbored *Pseudomonas aeruginosa* in their respiratory secretions in parallel to *S. agalactiae*. Diabetes as a clinical presentation is not unusual in older CF patients and *S. agalactiae* infections are more prevalent in diabetic patients [12]. However among the CF patients with *S. agalactiae* none had diabetes, when *S. agalactiae* was first isolated, even though one patient became diabetic 4 years later.

**Table 1:** Clinical characteristics of *S. agalactiae* (GBS) positive cystic fibrosis patients.

patient	Gender f(female) m (male)	Age at GBS isolation in years	ST of initial GBS isolate	Number of repeated GBS isolates available for analysis	STs of repeated isolates	Patient diabetic at GBS isolation	Visit due to exacerbation of symptoms (E) or routine control(R)	Concomitant pathogenic bacterial isolates
1	m	19	12	2	26/12	no	E/R/E	*S. aureus*
2	f	5	10	-	-	no	unknown	*M. catarrhalis*
3	m	24	1	-	-	no	E	*H. influenzae*
								*P. fluoreszenz*
4	f	39	1	-	-	no	E	*P. aeruginosa*
5	f	13	1	3	23/1/10	no	E/E/E/R	*S. aureus*
								*E. coli*
6	f	12	12	2	12/12	no	R/R/R	*S. aureus*
								*H. influenzae*
7	m	17	88	2	88/88	no	unknown	*S. aureus*
8	f	15	88	1	88	no	R/R	*S. aureus*
								*E. cloacae*
9	f	23	22	1	22	no	R/E	*S. aureus*
								*P. aeruginosa*
10	m	13	1	-	-	no	E	*S. aureus*
11	f	14	88	-	-	no	E	*S. aureus*
12	m	10	19	-	-	no	R	*P. aeruginosa*
								atypical Mycobacteria
13	m	20	19	-	-	no	E	*P. aeruginosa*
								*A. xylosoxidans*
14	m	20	103	1	103	no	R/R	-
15	m	26	19	-	-	no	E	*S. aureus*
16	f	10	8	-	-	no	R	*S. aureus*

### MLST sequence types and burst analysis

**Figure 1:**
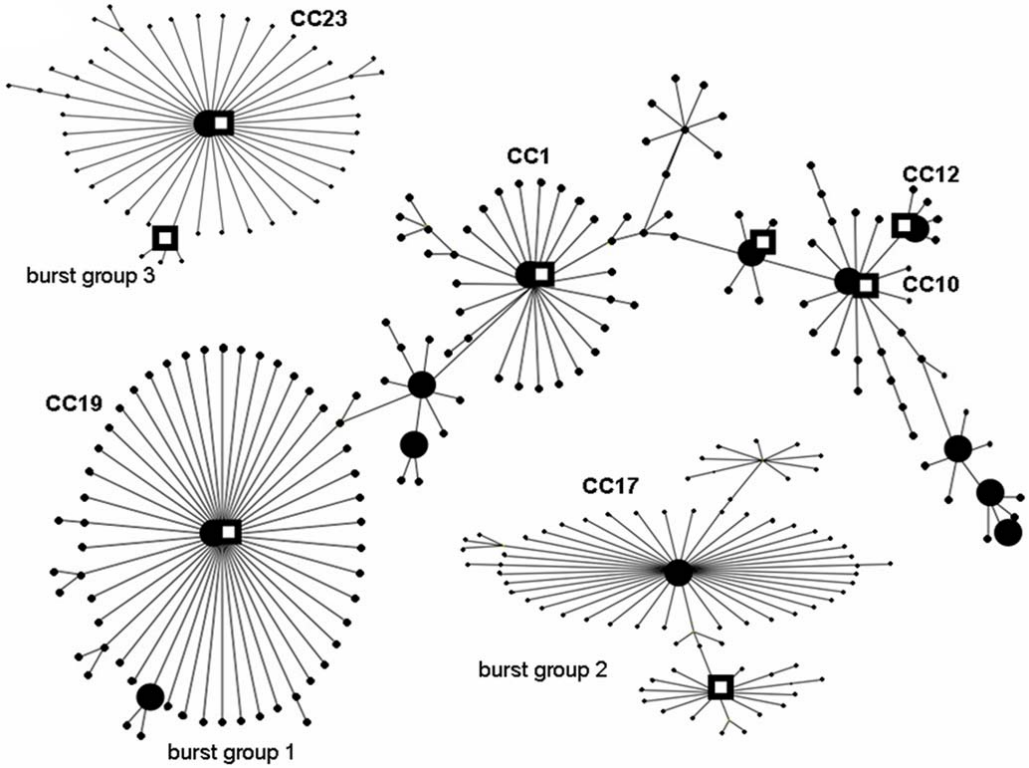
Population structure of *Streptococcus agalactiae*: Depicted are the three major recognized burst groups of *S. agalactiae*. Sequence types that vary by one allele in their MLST profiles (single locus variants) are arranged in circles around the primary founder sequence type. The population structure diagram was created based on the *S. agalactiae* MLST database as found under: http://eburst.mlst.net. Sequence types present in our collection of colonizing strains are depicted as closed circles, sequence types found in the *S. agalactiae* strains form CF patients are shown as open squares. The major clonal complexes (CC) are indicated in the picture.

Unequivocal MLST sequence types could be assigned to all of the 19 CF strains and to all of the 72 colonizing strains that were investigated in this study. Among the *S. agalactiae* strains from CF patients ten different MLST sequence types were observed (Table 2). This is a clear indication that we did not encounter the situation of a local outbreak. To identify clonal complexes and to reveal relationships among strains, the colonizing strains as well as the *S. agalactiae* strains isolated from CF patients were subjected to an analysis by the eBURSTV3 program. Most of the colonizing strains (66%) and 13 of 19 CF isolates belonged to BURST group 1 (Table 2 and Table 3), which represents the biggest clonal complex in the *S. agalactiae* population. Strains belonging to the largest 3 of the so far recognized BURST groups of *S. agalactiae* were present in the CF isolates as well as in the colonizing strains. The pattern observed for the colonizing strains did not appear to differ substantially from the pattern and clonal complexes that are present in the *S. agalactiae* MLST database, even though some STs were present only in colonizing or CF strains (Table 2 and Table 3). A graphic representation of the data is shown in Fig. 1.

**Table 2:** Molecular characterization of 19 unique *S. agalactiae* strains from CF patients.

Sequence type	Burst group	Number of isolates	serotype	Surface protein
1	1	3	V	Alp2/3
		1	VI	alpha C
8	1	1	Ib	alphaC
10	1	2	Ib	alpha C
12	1	1	Ib	alpha C
		1	II	alpha C
19	1	2	III	Rib
		1	NT*	Rib
22	2	1	NT*	alphaC
23	3	1	Ia	Epsilon
26	5	1	V	-
88	3	3	Ia	Alp2/3
103	7	1	Ia	alphaC

* Nontypable.

**Table 3:** Molecular characterization of 72 colonizing *S. agalactiae* strains from the urogenital and gastrointestinal tract.

Sequence type	Burst group	Number of isolates	serotype	Surface protein
1	1	6	V	Alp2/3
2	1	1	Ia	-
6	1	1	Ib	alpha C
7	1	1	Ia	alpha C
8	1	7	Ib	alpha C
10	1	2	Ia	alpha C
		1	Ib	alpha C
		4	II	alpha C
12	1	2	Ib	alpha C
		4	II	alpha C
17	2	9	III	Rib
19	1	2	II	Rib
		7	III	Rib
		1	III	Alp2/3
		1	III	-
		1	V	Rib
23	3	8	Ia	Epsilon
		1	Ia	Alp2/3
		2	III	Alp2/3
		1	III	Epsilon
28	1	2	II	Alp2/3
		2	II	-
		1	II	alpha C
41	1	1	V	Rib
196	1	1	IV	Epsilon
314	4	1	Ia	Epsilon
389	none	1	II	Alp2/3
		1	III	Rib

### Capsular serotype and surface protein distribution

In addition to the MLST analysis, all of the *S. agalactiae* strains included in this study were characterized by capsular serotyping and surface protein determination. Capsular serotypes could be obtained for 17 of the 19 CF strains and all of the colonizing strains. With the exception of fives strains (one CF and four colonizing isolates) all of the strains also harbored a gene for one of the following surface proteins: alpha C, Epsilon, Alp2/3 or Rib.

### Association of surface protein antigens with MLST sequence types

**Figure 2:**
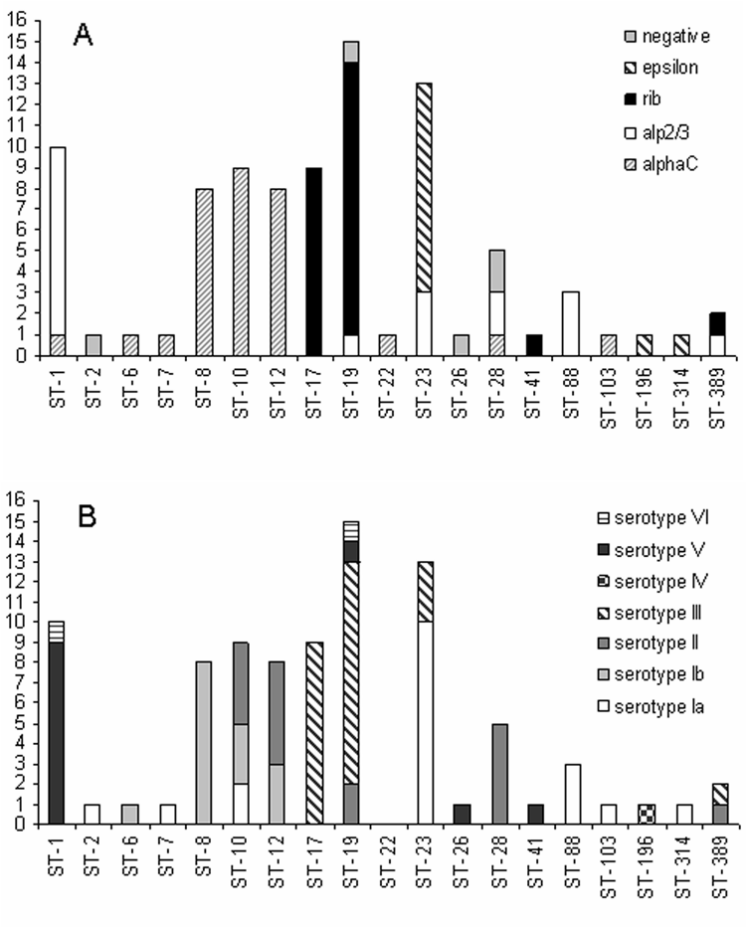
A: Association between surface proteins and sequence types. For each sequence type found in either respiratory strains from CF patients or colonizing strains, the number of isolates and the surface proteins of these strains are shown. Genes coding for alpha C, Epsilon, Rib or Alp2/3 were detected in the vast majority of strains, only five isolates failed to generate a PCR product with the specific primers. B: Association between serotypes and sequence types. For each sequence type found in either respiratory strains from CF patients or colonizing strains, the number of isolates and the serotypes of these strains are shown.

It has repeatedly been shown in epidemiologic investigations that a specific capsular serotype of *S. agalactiae* can harbor different surface protein antigens [10], [13]. To investigate if this is also true for MLST sequence types, we determined the surface antigen profile of the analyzed strains and compared it with the respective MLST sequence types. While the number of strains belonging to a specific sequence type in this investigation was of course limited, the association between sequence type and surface protein antigen appeared to be closer for some sequence types than the association between capsular serotype and surface proteins (Fig. 2). Among the ST-10 isolates from CF patients (n = 2) and the colonizing strains (n = 7), we detected three different serotypes (Ia, Ib and II) but all of the strains harbored the alpha C protein. However for the 5 colonizing ST-28 strains we saw a bigger variety of surface proteins than serotypes. Unfortunately the number of strains for each ST was too low to allow statistical analysis.

### Antimicrobal susceptibilty

CF patients are routinely and repeatedly treated with antibiotics for their respiratory infections. To investigate, if increased antibiotic resistance rates are observed in the CF patients that harbor *S. agalactiae* in respiratory secretions, MIC (minimal inhibitory concentration) values for a panel of different antibiotics were determined and MIC50 and MIC90 data is shown in Table 4. All of the strains were fully susceptible to penicillin. One of the CF isolates displayed high level gentamicin resistance with a MIC of ≥1024 mg/l. In addition a macrolide resistance was observed in 4 of 19 unique strains and 11 of 19 strains were resistant to doxycycline. Any further relevant resistance patterns could not be detected.

**Table 4:** Antimicrobial susceptibilities of *S. agalactiae* isolates from cystic fibrosis patients.

agent	MIC50 (mg/l)	MIC90 (mg/l)	Number of resistant or intermediate susceptible isolates
Penicillin	≤0.0625	≤0.0625	0/19
Ampicillin	≤0.125	≤0.125	0/19
Cefuroxime	≤0.125	≤0.125	0/19
Ceftriaxone	≤0.125	≤0.125	0/19
Erythromycin	0.5	>8	4/19
Clarithromycin	≤0.0625	>8	4/19
Clindamycin	0.125	>8	3/19
Doxycyclin	4	8	11/19
Rifampicin	0.125	0.125	0/19
Gentamicin	48	64	1/19*
Levofloxacin	0.75	1	0/19
Moxifloxacin	0.25	0.25	0/19
Vancomycin	0.5	0.5	0/19

* Depicted is the number of high level (≥512 mg/l) gentamicin resistant strains.

## Discussion

*S. agalactiae* is only rarely seen as a colonizing pathogen of the respiratory tract of healthy pediatric patients beyond the neonatal period. A comprehensive study screening for the presence of beta hemolytic streptococci found group B streptococci in less than 3% [1] of 184 healthy pediatric patients. But varying colonization rates have been published for throat cultures of healthy patients. One older US study showed a rate of 9% in a control group of students with a mean age of 23 [14]. This study however employed selective enrichment broth to optimize *S. agalactiae* recovery and was not performed in CF patients, which harbor a multitude of different bacteria in respiratory secretions. Studies on throat colonization in Europe revealed much lower rates [1], [2], [15]. Investigations of the microbiological species isolated from the respiratory tract of CF patients hardly ever reveal the presence of *S. agalactiae* [4]. Therefore, we were surprised to detect *S. agalactiae* in 16% in a population of 185 CF patients that were regularly screened during 2002–2008 for the presence of potential pathogens in their respiratory tract samples. Especially in view of the fact, that the samples were processed on regular blood agar plates, which grow a multitude of different organisms in CF patients. The detection rate of *S. agalactiae* in swabs increases about 100% if selective media for the cultivation of *S. agalactiae* are used [16]. Thus it is likely that the true isolation rate of *S. agalactiae* from the CF patients would be much higher, if antibiotic supplemented media that inhibit the growth of other pathogens were used for cultivation. This question however, could only be answered by a prospective investigation of respiratory tract samples from CF patients with liquid enrichment broth that optimizes the detection rate of *S. agalactiae*. Increased numbers of *S. agalactiae* infections have also been linked to diabetes [12] and CF patients may become diabetic during the course of their disease. But since none of the CF patients was diabetic at the time *S. agalactiae* was first isolated from their sputum, there is no indication that the high *S. agalactiae* isolation rates are due to an altered sugar metabolism in these patients.

*S. agalactiae* belongs to the group of beta hemolytic streptococci, which have a high virulence potential. In newborns and especially premature infants with reduced pulmonary surfactant it causes pneumonia and is a well recognized respiratory pathogen [17]. Surfactant associated protein A (SP-A) is the most abundant pulmonary surfactant protein [18] and its importance for *S. agalactiae* infections has been demonstrated in animal models [19]. In SP-A deficient knock out mice the clearance of intratracheally administered *S. agalactiae* is delayed. Interestingly the concentration of SP-A can be reduced in brocheo alveolar lavage fluid from CF patients [20], [21]. Older children and adult CF patients show consistently decreased levels of SP-A, in contrast to young children with CF. In our investigation, only one of the CF patients was younger than 10 years at the time *S. agalactiae* was first isolated from the sputum. Moreover the median age of CF patients with *S. agalactiae* isolates was 3 and 6 years older than the *S. agalcatiae* negative CF patients from the respective clinic. A finding which supports the hypothesis, that altered surfactant properties in this age group could be responsible for the emergence of *S. agalactiae* in the respiratory tract. It could also explain, why respiratory *S. agalactiae* isolates have previously not been reported in CF patients. Due to improved therapeutic regimens for CF patients in recent years, more than 35% of CF patients are now older than 18 years. Whereas in the 60's the predicted mean survival was only about 10 years [22]. But since we did not investigate the SP-A levels of the patients which harbor *S. agalactiae*, this is only a speculation that may be the target of further investigations.

To rule out a local outbreak among the CF patients in our area, 29 *S. agalactiae* strains from CF patients were collected and analyzed by molecular subtyping. Among these strains 19 unique isolates were found and 10 strains that were identical to previous isolates (Table 1). The presence of many different sequence types in the CF isolates (Table 2) shows that these strains are heterogeneous. We found no indication for a spread of a single *S. agalactiae* clone among the patients. Despite the limited number of strains from CF patients that we had for analysis, it is striking to see, that serotype III was rarely isolated in CF patients. This is in contrast to other German studies, in which serotype III isolates were most prevalent in invasive neonatal strains (65%) [23] as well as in colonizing strains (28%), obtained from adult women [8]. Analysis of the distribution of surface proteins among the CF strains revealed a similar pattern. As expected, the surface protein Rib was present in the serotype III strains we analyzed. Especially striking was the absence of any ST-17 strains in the samples from the CF patients. ST-17 serotpye III strains have been described as hypervirulent isolates in many different studies, and have a strong association with neonatal invasive disease and meningitis [24], [25]. In contrast to the total lack of ST-17 strains in CF patients, nine isolates of 72 from the German colonizing strains were ST-17 strains (Table 3), representing the third most frequent ST, an indication that ST-17 strains are present in considerable numbers in the pool of colonizing strains.

MLST characterization of *S. agalactiae* isolates from Germany has not been performed and published previously. Therefore in addition to the MLST analysis of the CF strains, we determined the MLST profiles of 72 colonizing *S. agalactiae* isolates, which were collected recently, as a reference population [8]. The MLST pattern we found in our collection of colonizing strains compares well to the MLST profiles seen in colonizing strains from other countries [24], [25]. The great majority of the MLST sequence types that we determined in the CF isolates, were also present in the German colonizing strains, indicating that the respiratory CF strains and the urogenital colonizing strains belong to the same population. In order to correlate the MLST sequence types with known molecular markers of *S. agalactiae*, the CF isolates as well as the colonizing strains from the urogenital tract of healthy patients were characterized by serotyping and surface protein determination. While it is well-known, that a correlation between *S. agalactiae* serotypes and surface protein antigens exists [13], a detailed analysis of the association of MLST sequence types and surface proteins has not been published. Since there are many more MLST sequence types than recognized *S. agalactiae* serotypes, it was not surprising to see, that for most of the sequence types, only a single type of surface protein was found in our investigation. However for sequence types with many isolates like ST -1, ST- 19, ST-23 and ST-28, we were able to detect strains with varying surface proteins. For selected sequence types, like ST-10 and ST-12 the association between MLST profiles and surface proteins appeared to be closer than the association between sequence type and serotype (Tables 2 and 3). In ST-10 and ST-12 isolates, different serotypes were detected in one MLST sequence type, but all of the strains belonging to that specific sequence type harbored the same surface protein (Table 2 and 3). This was however not true for all of the sequence types that we found, since in ST-28 only serotype II was observed, but alpha C protein as well as Alp2/3 were detected in these strains. Overall the number of isolates we had for analysis is too small to reach any definite conclusions about the association of sequence types and surface protein antigens.

During the course of many years CF patients are repeatedly treated with anitibiotics to limit respiratory infections. Under these conditions exposure of bacterial strains to various antibiotics for a prolonged time is quite common, in contrast to the pregnant and mostly healthy patients, which are usually screened for the presence of *S. agalactiae* colonization. For other bacterial pathogens high levels of antibiotic resistance rates have been reported in CF patients [26]. In our collection of *S. agalactiae* isolates from CF patients we were also able to observe an unusual resistance pattern. One of the strains exhibited a high level gentamicin resistance. This type of resistance is not common in *S. agalactiae* but has previously been reported [27], [28] in a few isolates. Resistance rates to macrolides were found to be 21%, which is comparable to the macrolides resistance rates of recent studies for *S. agalactiae* [29], [30]. In these investigations rates between 11% and 38% were published.

In conclusion, we report the detection of *S. agalactiae* in a considerable proportion of respiratory samples of CF patients. Detailed molecular analysis of the strains did not reveal a local outbreak. *S. agalactiae* positive patients were several years older than a reference population of CF patients, which is consistent with the hypothesis, that an altered surfactant composition in this age group supports *S. agalactiae* growth. A condition, which may help the adaptation of *S. agalactiae* to a novel, not yet recognized host environment.
